# Supplementary material for: Excellent energy storage properties in lead-free ferroelectric ceramics via heterogeneous structure design
Source: Nat Commun. 2025 Feb 14;16:1633. doi: 10.1038/s41467-025-56767-0 (PMC11828929; doi:10.1038/s41467-025-56767-0)
Supplement: Supplementary file 1 — Supplementary Information [file 41467_2025_56767_MOESM1_ESM.pdf]

Supplementary Information

Excellent energy storage properties in lead-free ferroelectric ceramics via heterogeneous structure design

Qizhen Chai[1], Zhaobo Liu[2], Zhongqi Deng[3], Zhanhui Peng[1], Xiaolian Chao[1], Jiangbo Lu[3,*], Houbing Huang[2,*], Shujun Zhang[4,*], Zupei Yang[1,*]

* Corresponding authors, E-mail addresses: jblu10@snnu.edu.cn (J. Lu); hbhuang@bit.edu.cn (H. Huang); shujun@uow.edu.au (S. Zhang); yangzp@snnu.edu.cn (Z. Yang)

This PDF file includes:

Table S1 to S5

Fig. S1 to S12

Experimental

References

Supplementary Table 1 Enhanced energy storage properties of BNT-based bulk ceramics through various optimization strategies[1-14].

Composition	Optimization Strategy	W_{rec} (J cm^{-3})	η (%)	E_b (kV cm^{-1})
$0.8Bi_{0.395}Na_{0.325}Sr_{0.245}TiO_3$-$0.2Ca_{0.7}La_{0.2}TiO_3$	phase structure engineering	8.3	80	660
$0.92(0.65Na_{0.5}Bi_{0.5}TiO_3$-$0.35Sr_{0.85}Bi_{0.1}TiO_3)$-$0.08Bi(Mg_{0.5}Sn_{0.5})O_3$	construct Sr^{2+}-Sr^{2+}, induce PNRs, and implement viscous polymer process	7.5	85	440
$0.85(0.94Bi_{0.5}Na_{0.5}TiO_3$-$0.06BaTiO_3)$-$0.15BiMg_{2/3}Nb_{1/3}O_3$	construct the coexistence of polymorphic PNRs	6.3	79.6	420
$0.6Bi_{0.5}Na_{0.5}TiO_3$-$0.4(0.7SrTiO_3$-$0.3Bi_{0.5}Li_{0.5}TiO_3)$+0.5 at.%$Nb_2O_5$	construct PNRs and nanodomains, ultrafine grains and intrinsic conduction	8.63	89.6	520
$(Bi_{0.5}Na_{0.5})_{0.7}Sr_{0.3}Ti_{0.8}(Sc_{0.5}Ta_{0.5})_{0.2}O_3$	induce the evolution of oxygen vacancies and local polar phases	12.2	85.9	590
$(Bi_{0.47}Sm_{0.03}Na_{0.42})_{0.94}Ba_{0.06}TiO_3$	A-site defect engineering	4.62	79.1	290
$0.88(0.65Bi_{0.5}Na_{0.5}TiO_3$-$0.35SrTiO_3)$-$0.12Bi(Mg_{0.5}Hf_{0.5})O_3$	increase band gap, refine grain size and nano-domains	5.59	85.3	390
$0.9(Bi_{0.5}Na_{0.5})_{0.65}Sr_{0.35}TiO_3$-$0.1Bi(Mg_{0.5}Zr_{0.5})O_3$	induce the quenched random field and PNRs; refine grain size	8.46	85.9	522
$0.62(0.94Na_{0.5}Bi_{0.5}TiO_3$-$0.06BaTiO_3)$-$0.38Ca_{0.7}La_{0.2}TiO_3$	introduce super-paraelectric state; refine grain size; adjust activation energy differences	15.1	82.4	640
$0.7Bi_{0.47}Na_{0.47}Ba_{0.06}TiO_3$-$0.3Sr_{0.7}La_{0.2}Ta_{0.2}Ti_{0.75}O_3$	entropy engineering	15.48	90.02	710
$0.8Bi_{0.5}Na_{0.5}TiO_3$-$0.2Ba_{0.7}Sr_{0.3}Zr_{0.8}Sn_{0.2}O_3$	construct multiphase structures	7.4	89	400
$0.85Bi_{0.5}Na_{0.5}TiO_3$-$0.15AgNb_{0.5}Ta_{0.5}O_3$	phase structure and defect engineering	6.6	72	510
$Na_{0.36}Bi_{0.388}Ca_{0.238}TiO_3$	introduce linear $Ca_{0.85}Bi_{0.1}TiO_3$ additive	7.13	83	420
$Bi_{0.2}Na_{0.2}Ba_{0.2}Sr_{0.2}Ca_{0.2}TiO_3$+5 mol%$Li_2CO_3$	high-entropy	10.7	89	640

Supplementary Table 2 Enhanced energy storage properties of BF-based bulk ceramics through various optimization strategies[15-23].

Composition	Optimization Strategy	W_{rec} (J cm^{-3})	η (%)	E_b (kV cm^{-1})
$(Bi_{0.5}Ba_{0.1}Sr_{0.1}Ca_{0.2}Na_{0.1})(Fe_{0.5}Ti_{0.3}Zr_{0.1}Nb_{0.1})O_3$	high-entropy	13.3	78	664
$0.46Bi_{1.02}FeO_3$-$0.29BaTiO_3$-$0.25Bi_{0.5}Na_{0.5}TiO_3$-$0.5Nb_2O_5$	increase the local structure disorder	3.9	80	250
$0.7(0.67BiFeO_3$-$0.33BaTiO_3)$-$0.3Ca_{0.85}Bi_{0.05}Sm_{0.05}TiO_3$	increase disorder at the A-site and reducing grain size	5.26	82.40	300
$0.5BiFeO_3$-$0.4BaTiO_3$-$0.1CaHfO_3$	induce cubic phase, reduce grain size, and enhance electrical resistivity	4.7	79	410
$0.88(0.67BiFeO_3$-$0.33BaTiO_3)$-$0.12Na_{0.73}Bi_{0.09}NbO_3$	tape-casting processing	5.57	83.80	410
$0.57BiFeO_3$-$0.33BaTiO_3$-$0.1NaNbO_3$	integrate high polarization, wide band gaps, and heterogeneous nanodomains	8.12	90	360
$(0.67BiFeO_3$-$0.33BaTiO_3)$-$0.15Sr(Nb_{0.5}Al_{0.5})O_3$	crossover relaxor ferroelectric state	3.95	85.9	300
$0.35BiFeO_3$-$0.65SrTiO_3$	composition and structural modification	8.4	90	750
$0.53BiFeO_3$-$0.3Ba(Hf_{0.05}Ti_{0.95})O_3$-$0.17NaTaO_3$	widen the band gap, increase resistivity, and enhance relaxor behavior	6.3	86.6	425

Supplementary Table 3 Enhanced energy storage properties of BT-based bulk ceramics through various optimization strategies[24-32].

Composition	Optimization Strategy	W_{rec} (J cm^{-3})	η (%)	E_b (kV cm^{-1})
$0.85(0.8BaTiO_3\text{-}0.2(Bi_{0.5}Na_{0.5})TiO_3)\text{-}0.15CaZrO_3$	heterostructure design (coexisting R-T PNRs)	9.04	87.2	540
$0.91(Ba_{0.8}Sr_{0.2})TiO_3$ $\text{-}0.09Bi(Zn_{2/3}Nb_{1/3})O_3$	a synergistic two-step strategy (composition and viscous polymer processing)	5.16	82.3	540
$2/9BiFeO_3\text{-}1/3BaTiO_3\text{-}2/9Bi_{0.5}Na_{0.5}TiO_3\text{-}2/9NaNbO_3$	a highly polarizable concentrated dipole glass	15.9	93.3	670
$0.8(0.75BaTiO_3\text{-}0.25Na_{0.5}Bi_{0.5}TiO_3)\text{-}0.2(Sr_{0.7}Bi_{0.2})(Mg_{1/3}Ta_{2/3})O_3$	improve relaxation behavior and insulation performance	7.12	90	720
$1/3BaTiO_3\text{-}1/3Bi_{0.5}Na_{0.5}TiO_3\text{-}1/3NaNbO_3$	construct local diverse polarization	10.59	87.6	550
$0.6BaTiO_3\text{-}0.4Bi(Mg_{1/2}Ti_{1/2})O_3$	nano-scale polarization mismatch and reconstruction	4.49	93	340
$0.85(Ba_{0.8}Sr_{0.2})TiO_3\text{-}0.15Bi(Mg_{0.5}Zr_{0.5})O_3$	induce PNRs and use viscous polymer process	10.3	88	720
$Ba_{0.82}Bi_{0.12}TiO_3$	local structure design	10.1	90	700
$0.85BaTiO_3\text{-}0.15(Bi_{0.5}Na_{0.5})(Zn_{1/3}Nb_{2/3})O_3$	control inhomogeneous polarization configuration	8.6	97.4	460
	inhomogeneous polarization configuration and high-energy ball milling	11.6	96.1	580

Supplementary Table 4 Enhanced energy storage properties of KNN-based bulk ceramics through various optimization strategies[33-41].

Composition	Optimization Strategy	W_{rec} (J cm^{-3})	η (%)	E_b (kV cm^{-1})
$0.85K_{0.5}Na_{0.5}NbO_3$-$0.15Bi(Zn_{2/3}Ta_{1/3})O_3$	drive a specific temperature region and implement a repeated rolling process	6.7	92	600
$0.85K_{0.5}Na_{0.5}NbO_3$-$0.15Bi(Ni_{0.5}Zr_{0.5})O_3$	tailor grain size to submicron scale and adjust the temperature range	8.09	88.46	870
$0.90K_{0.5}Na_{0.5}NbO_3$-$0.10Bi(Zn_{2/3}(Nb_{0.85}Ta_{0.15})_{1/3})O_3$	induce polar nano-regions and reduce grain size	7.4	78	800
$[(K_{0.2}Na_{0.8})_{0.8}Li_{0.08}Ba_{0.02}Bi_{0.1}](Nb_{0.68}Sc_{0.02}Hf_{0.08}Zr_{0.1}Ta_{0.08}Sb_{0.04})O_3$	high-entropy	10.06	90.8	740
$(Na,K)(Sb,Nb)O_3$-$SrZrO_3$-$(Bi_{0.5}Na_{0.5})ZrO_3$	engineer multiple local symmetries; use spark plasma sintering	13.1	90	740
$0.825(K_{0.5}N_{a0.5})NbO_3$-$0.175Sr(Sc_{0.5}Nb_{0.5})O_3$	introduce nanodomains, increase band gap energy, and reduce grain size	2.67	60	395
$0.94(K_{0.5}Na_{0.5})NbO_3$-$0.06Sr_{0.7}La_{0.2}ZrO_3$	refine the grain size, introduce PNRs, and induce a pseudo-cubic phase	5.3	71	490
0.25 mol%Er-$K_{0.5}Na_{0.5}NbO_3$-$Sr_1Ba_{0.5}TiO_3$	A-site non-stoichiometric defect engineering	3.42	53.5	320
$0.91K_{0.5}Na_{0.5}NbO_3$-$0.09SrZrO_3$	refine grain size	2.81	80	370

Relaxor ferroelectric ceramics, in contrast to typical ferroelectrics, exhibit slimer *P-E* loops, which facilitates the simultaneous enhancement of W_{rec} and η[42-44]. Among these, BNT, BF, BT, and KNN-based lead-free ceramics have emerged as research hotspots. Tables 1-4 provide a comprehensive summary of the majority of BNT, BF, BT, and KNN-based relaxor ferroelectric ceramics that have been published in prestigious journals over the past five years. Although various methods have demonstrated effectiveness in improving energy storage properties, the currently achievable synergistic values of W_{rec} and η remain unsatisfactory due to the inherent negative correlation between E_b and ΔP, especially for simple solid-state reactions and/or chemical compositions.

Supplementary Table 5 A comparative analysis of mechanisms in heterogeneous structures, high-entropy effects, domain engineering, defect engineering, and grain size engineering[3-6,10,14,34].

Mechanism	Description	Key Contributions to Energy Storage	Unique Features
**Heterogeneous Structure** (dominant)	Coexistence of rhombohedral and/or orthorhombic, as well as tetragonal polar nanoregions within a cubic matrix	Enhance polarization while minimizing hysteresis, thereby elevating energy density and enhancing efficiency	Introduce polymorphic nanodomains
**High-Entropy engineering** (negligible)	Incorporation of multiple principal elements in approximately equal proportions	Stabilize multiple phases, enhance thermal and chemical stability, and broaden polarization, thereby enhancing energy storage performance	Composition of multiple elements and unique microstructure
	$\Delta S_{config} \geq 1.61R$		
**Domain Engineering** (significant)	Manipulation of domain structures in ferroelectric materials	Optimize domain wall motion and switching dynamics, thereby improving energy density and efficiency	Control domain sizes, orientation, and switching behavior
**Defect Engineering** (beneficial)	Introduce and regulate defects in materials	Modify electronic and ionic conductivity	Act as charge traps or enhance carrier mobility
		Refine the grain size and domain size due to vacancy-related defect pinning, thereby enhancing overall energy storage performance	Regulate the oxygen vacancies
**Grain Size Engineering** (significant)	Refinement of grain size and regulation distribution in polycrystalline materials	Hinder domain growth, improve the breakdown strength, thereby elevating the energy storage density	Refine the grain size to submicron level

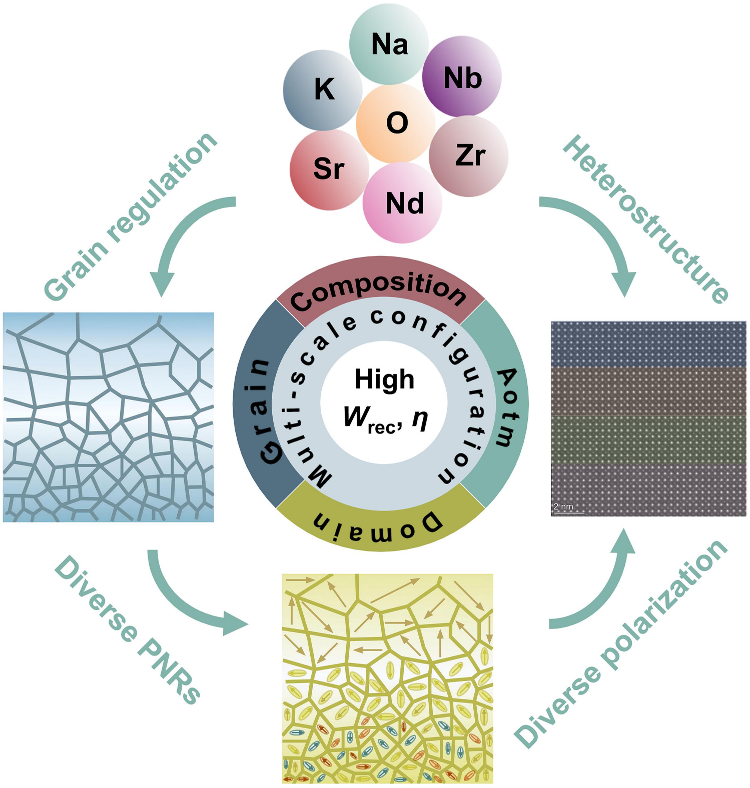

Supplementary Fig. 1 Schematic diagram for realizing enhanced energy storage performance via multi-scale optimization.

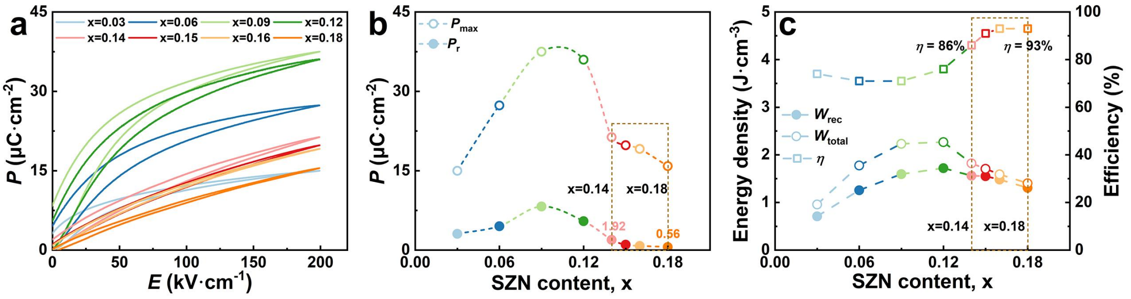

Supplementary Fig. 2 Energy storage properties of (1-*x*)KNN-*x*SNZ ceramics under an electric field of 200 kV cm^{-1}. a Ambient-temperature *P-E* loops and **b** P_{max} and P_r. **c** Calculated values of W_{rec}, W_{total}, and η.

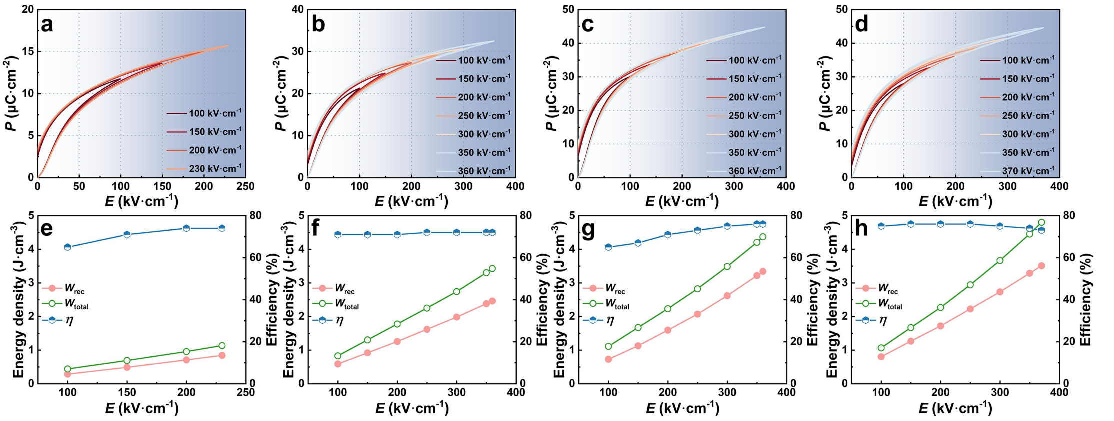

Supplementary Fig. 3 Energy storage properties of (1-*x)*KNN-*x*SNZ ceramics measured under various electric fields. Ambient-temperature *P-E* loops of **a** $x = 0.03$, **b** $x = 0.06$, **c** $x = 0.09$, **d** $x = 0.12$. Composition-dependent energy storage parameters (W_{rec}, W_{total}, and η) of **e** $x = 0.03$, **f** $x = 0.06$, **g** $x = 0.09$, **h** $x = 0.12$, all samples used in this study are 0.1 mm in thickness with electrode area of 0.785 mm^2.

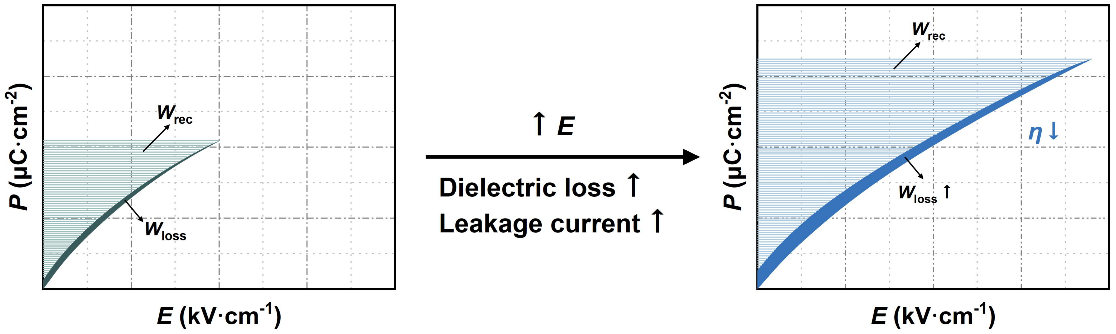

Supplementary Fig. 4 Schematic of the relationship between η and E.

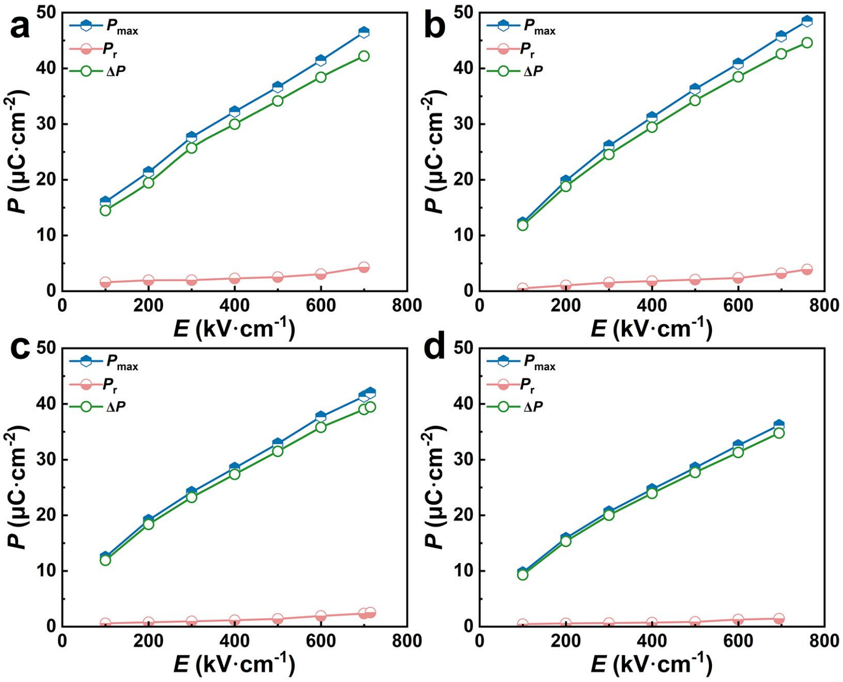

Supplementary Fig. 5 Polarization properties of (1-*x*)KNN-*x*SNZ ceramics. **a** $x = 0.14$, **b** $x = 0.15$, **c** $x = 0.16$, **d** $x = 0.18$.

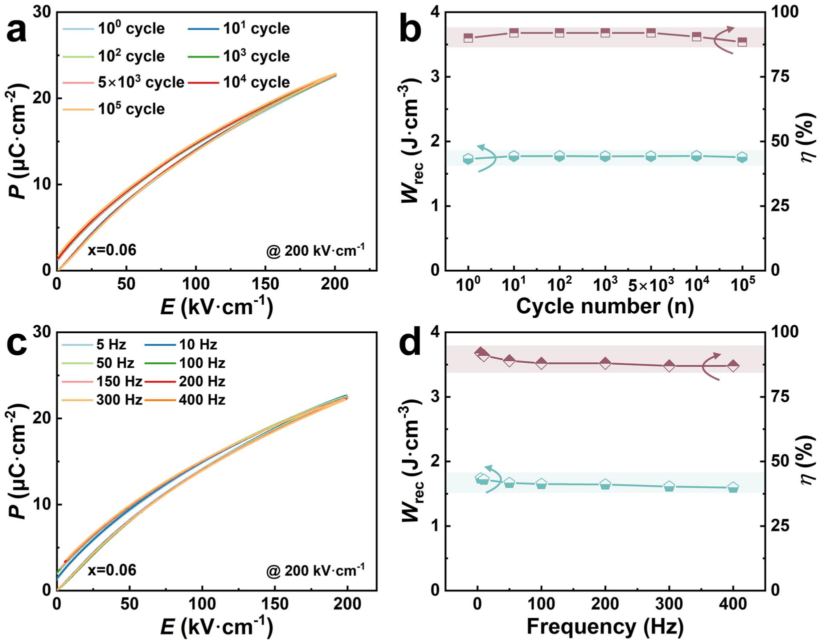

Supplementary Fig. 6 Reliability and stability measurements of energy-storage performance at an electric field of 200 kV cm^{-1} of the $x = 0.15$ ceramic. Fatigue resistance of **a** *P-E* loops, and **b** W_{rec} and η values with respect to cycling numbers. **c** *P-E* loops, and **d** W_{rec} and η values with

respect to frequency. The *P-E* loops, which show negligible changes over 10^5 cycles and frequencies ranging from 5 to 400 Hz at 200 kV cm^{-1}, as shown in **a** and **c**, confirm the superior cycling reliability (W_{rec} ~1.77 ± 0.02 J cm^{-3}, η ~90.0 ± 2.0%) and frequency stability (W_{rec} ~1.67 ± 0.07 J cm^{-3}, η ~89.5 ± 2.5%) for x = 0.15 ceramics, as illustrated in **b** and **d**, respectively.

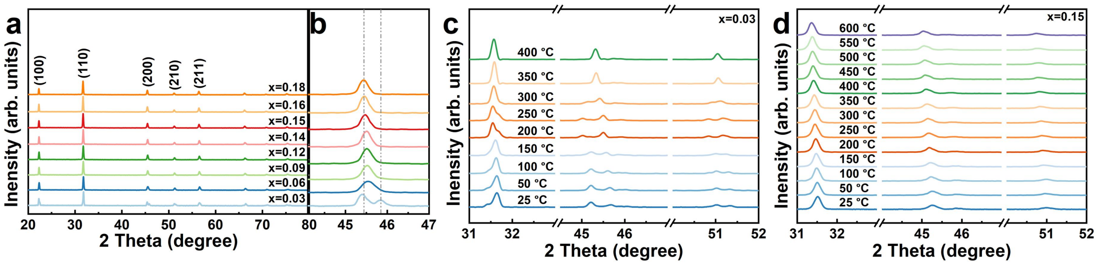

Supplementary Fig. 7 XRD patterns of (1-*x*)KNN-*x*SNZ ceramics. a XRD patterns measured at ambient temperature. **b** Enlarged view of 2θ from 44° to 47°. Temperature-dependent XRD patterns of **c** x = 0.03 and **d** x = 0.15 ceramics.

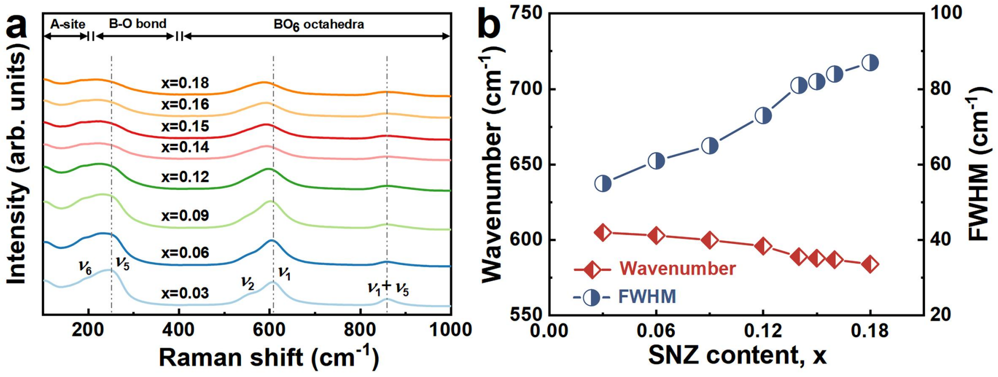

Supplementary Fig. 8 The evolution of Raman spectra of (1-*x*)KNN-*x*SNZ ceramics. a Raman spectra measured at ambient temperature. **b** Composition-dependent wavenumber and FWHM of the v_1 mode.

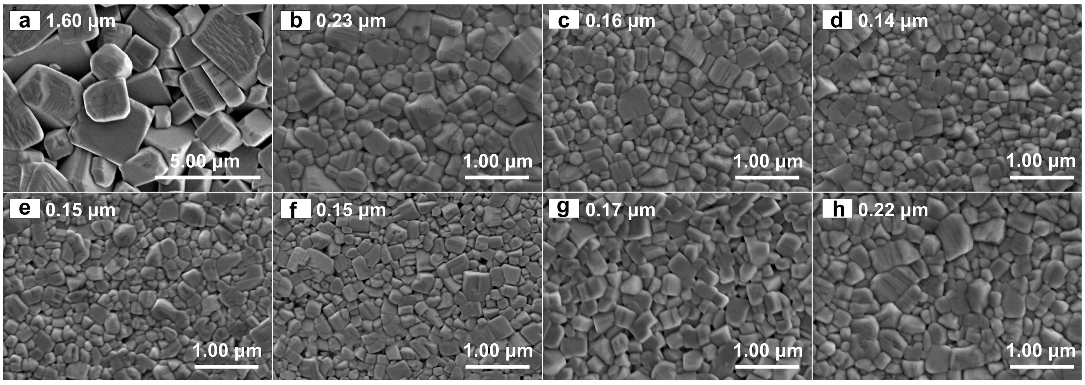

Supplementary Fig. 9 FE-SEM images of (1-*x*)KNN-*x*SNZ ceramics. **a** $x = 0.03$, **b** $x = 0.06$, **c** $x = 0.09$, **d** $x = 0.12$, **e** $x = 0.14$, **f** $x = 0.15$, **g** $x = 0.16$, **h** $x = 0.18$.

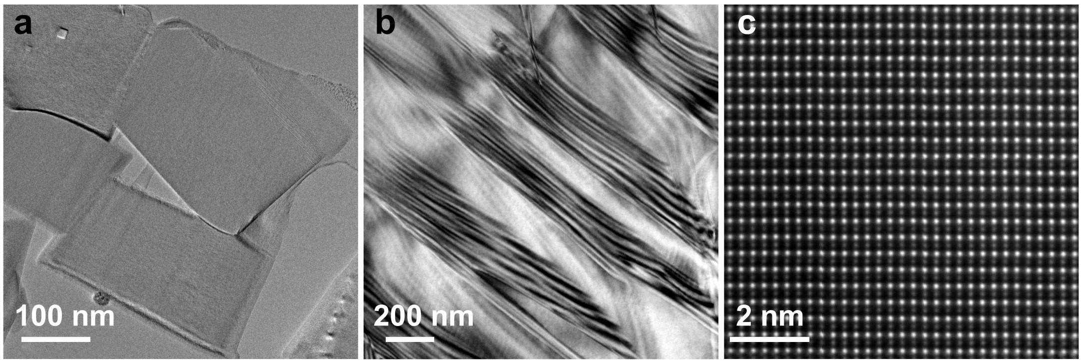

Supplementary Fig. 10 TEM images of (1-*x*)KNN-*x*SNZ ceramics. **a** $x = 0.15$, **b** pure KNN. **c** Atomic resolution HAADF-STEM image along [110]c of $x = 0.15$ ceramic.

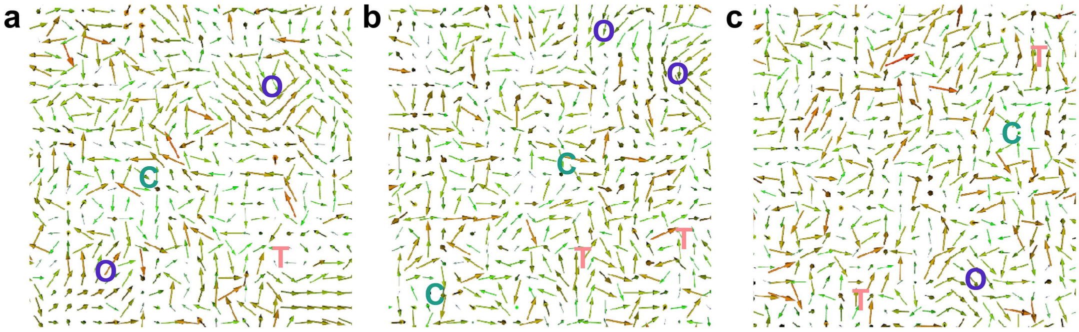

Supplementary Fig. 11 Simulation of the two-dimensional domain structure of (1-*x*)KNN-*x*SNZ ceramics. **a** $x = 0.12$, **b** $x = 0.15$, **c** $x = 0.18$, exhibiting the O and T nanodomains in the C matrix.

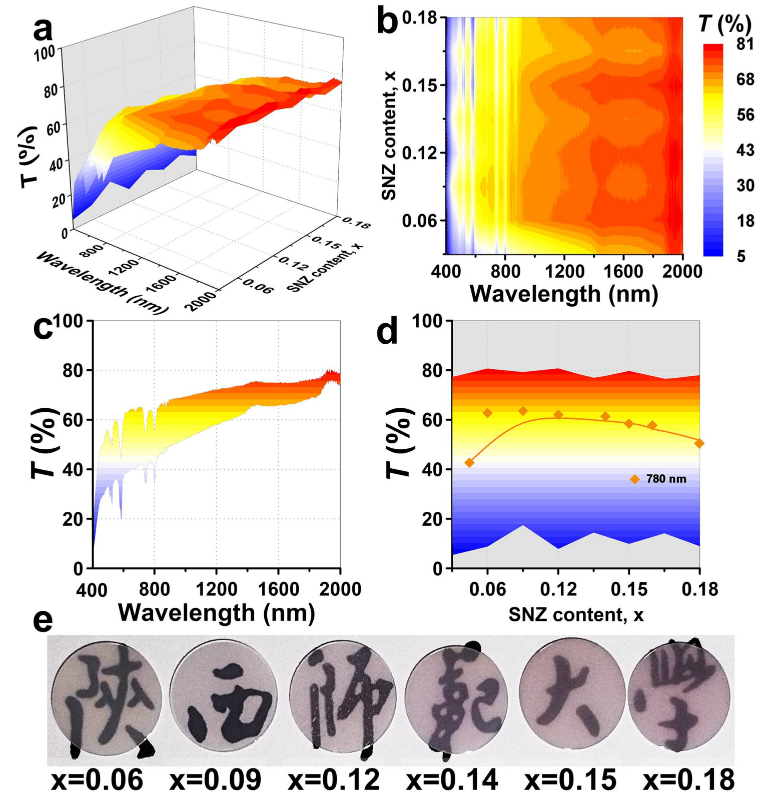

Supplementary Fig. 12 Optical transmittance spectra and visual photograph of (1-*x*)KNN-*x*SNZ ceramics. **a** 3D plots, **b** top view, **c** left view, and **d** front view. **e** Visual photograph with a thickness of ~0.3 mm.

Experimental

Phase-Field Simulations: To reveal the temporal evolution of the polarization field and the domain structure, we employed the time-dependent Ginzburg-Landau (TDGL) equation[45-48],

$$\frac{\partial P_i(r,t)}{\partial t} = -L\frac{\delta F}{\delta P_i(r,t)'}(i=1,2,3) \tag{S1}$$

where $P_i(r, t)$ is the polarization, r is the space position, t is time, L is the kinetic coefficient, and F is the total free energy of the system.

The total free energy of the system includes the bulk free energy, the gradient energy, the elastic energy, and the electrostatic energy:

$$F = \int \left(f_{bulk} + f_{grad} + f_{ela} + f_{elec}\right)dV \tag{S2}$$

where V is the system volume, f_{bulk} represents the Landau bulk free-energy density, f_{grad} is the gradient energy density, f_{ela} is the elastic energy density, and f_{elec} is the electrostatic energy density. The bulk free-energy density for a stress-free ferroelectric can be expressed as:

$$f_{bulk} = a_1 P_i^2 + a_{11} P_i^4 + a_{12} P_i^2 P_j^2 + a_{111} P_i^6 + a_{112} P_i^4 P_j^2 + a_{123} P_i^2 P_j^2 P_k^2 \quad \text{(S3)}$$

where a_1, a_{11}, a_{12}, a_{111}, a_{112}, a_{113} are Landau energy coefficients, whose values depend on concentration *c* and temperature *T* and determine the thermodynamic behaviors of bulk phases. The gradient energy density is related to the contribution of the domain wall to the total free energy,

$$f_{grad} = \frac{1}{2} G_{ijkl} P_{i,j} P_{k,l} \quad \text{(S4)}$$

where $P_{i,j} = \partial P_i / \partial x_j$, G_{ijkl} is the gradient energy coefficient. The homologous elastic energy density can be obtained as:

$$f_{elas} = \frac{1}{2} C_{ijkl} \left(\varepsilon_{ij} - \varepsilon_{ij}^0 \right) \left(\varepsilon_{kl} - \varepsilon_{kl}^0 \right) \quad \text{(S5)}$$

where C_{ijkl} is the elastic stiffness tensor, ε_{ij} is the total strain, ε_{ij}^0 is the electrostrictive stress-free strain, for a given domain structure, the electrostatic energy density is calculated by

$$f_{elec} = f_{dep} + f_{appel} \quad \text{(S6)}$$

$$f_{dep} = -\frac{1}{2} \varepsilon_0 \varepsilon_r E_i E_j \quad \text{(S7)}$$

$$f_{appel} = -E_i P_i \quad \text{(S8)}$$

where ε_0 is the vacuum conductivity, ε_r is the dielectric constant, and E_i is the local electric field.

Supplementary References

1. Tang, L. et al. Giant energy storage density with antiferroelectric-like properties in BNT-based ceramics via phase structure engineering. *Small* **19**, 2302346 (2023).

2. Wang, W. et al. Enhancing energy storage performance in $Na_{0.5}Bi_{0.5}TiO_3$-based lead-free relaxor ferroelectric ceramics along a stepwise optimization route. *J. Mater. Chem. A* **11**, 2641-2651 (2023).

3. Guo, B. et al. Energy storage performance of $Na_{0.5}Bi_{0.5}TiO_3$ based lead-free ferroelectric ceramics prepared via non-uniform phase structure modification and rolling process. *Chem. Eng. J.* **420**, 130475 (2021).

4. Long, C. et al. Excellent energy storage properties with ultrahigh W_{rec} in lead-free relaxor ferroelectrics of ternary $Bi_{0.5}Na_{0.5}TiO_3$-$SrTiO_3$-$Bi_{0.5}Li_{0.5}TiO_3$ via multiple synergistic optimization. *Energy Storage Mater*. **65**, 103055 (2024).

5. He, B. et al. Excellent energy storage performance of $(Sc_{0.5}Ta_{0.5})^{4+}$ modified $(Bi_{0.5}Na_{0.5})TiO_3$-based ceramics modulated by the evolution of polar phases. *J. Mater. Chem. A* **11**, 14169-14179 (2023).

6. Shen, Y. et al. Constructing novel binary $Bi_{0.5}Na_{0.5}TiO_3$-based composite ceramics for excellent energy storage performances via defect engineering. *Chem. Eng. J.* **439**, 135762 (2022).

7. Zhu, X. et al. Enhanced energy storage performance of $0.88(0.65Bi_{0.5}Na_{0.5}TiO_3$-$0.35SrTiO_3)$-$0.12Bi(Mg_{0.5}Hf_{0.5})O_3$ lead-free relaxor ceramic by composition design strategy. *Chem. Eng. J.* **437**, 135462 (2022).

8. Zhu, X. et al. Ultrahigh energy storage density in $(Bi_{0.5}Na_{0.5})_{0.65}Sr_{0.35}TiO_3$-based lead-free relaxor ceramics with excellent temperature stability. *Nano Energy* **98**, 107276 (2022).

9. Cao, W. et al. Interfacial polarization restriction for ultrahigh energy-storage density in lead-free ceramics. *Adv. Funct. Mater.* **33**, 2301027 (2023).

10. Duan, J. et al. High-entropy superparaelectrics with locally diverse ferroic distortion for high-capacitive energy storage. *Nat. Commun.* **15**, 6754 (2024).

11. Huang, Y. et al. Ultrahigh energy storage density, high efficiency and superior thermal stability in $Bi_{0.5}Na_{0.5}TiO_3$-based relaxor ferroelectric ceramics via constructing multiphase structures. *J. Mater. Chem. A* **11**, 7987-7994 (2023).

12. Che, Z. et al. Phase structure and defect engineering in $(Bi_{0.5}Na_{0.5})TiO_3$-based relaxor antiferroelectrics toward excellent energy storage performance. *Nano Energy* **100**, 107484 (2022).

13. Chen, P. et al. Outstanding energy-storage and charge-discharge performances in $Na_{0.5}Bi_{0.5}TiO_3$ lead-free ceramics via linear additive of $Ca_{0.85}Bi_{0.1}TiO_3$. *Chem. Eng. J.* **435**, 135065 (2022).

14. Guo, J. et al. Multi-symmetry high-entropy relaxor ferroelectric with giant capacitive energy storage. *Nano Energy* **112**, 108458 (2023).

15. Wu, J. et al. High energy storage performance in $BiFeO_3$-based lead-free high-entropy ferroelectrics. *Small* **20**, 2400997 (2024).

16. Wang, M. et al. Energy storage properties under moderate electric fields in $BiFeO_3$-based lead-free relaxor ferroelectric ceramics. *Chem. Eng. J.* **440**, 135789 (2022).

17. Liu, S. et al. Achieving excellent energy storage performance at moderate electric field in $Ca_{0.85}Bi_{0.05}Sm_{0.05}TiO_3$-modified $BiFeO_3$-based relaxor ceramics via multiple synergistic design. *Chem. Eng. J.* **470**, 144086 (2023).

18. Zhao, J. et al. Superior energy storage performance of $BiFeO_3$-$BaTiO_3$-$CaHfO_3$ lead-free ceramics. *J. Mater. Chem. A* **12**, 5261-5268 (2024).

19. Yan, F. et al. Optimization of polarization and electric field of bismuth ferrite-based ceramics for capacitor applications. *Chem. Eng. J.* **417**, 127945 (2021).

20. Qi, H. et al. Superior energy-storage capacitors with simultaneously giant energy density and efficiency using nanodomain engineered $BiFeO_3$-$BaTiO_3$-$NaNbO_3$ lead-free bulk ferroelectrics. *Adv. Energy Mater.* **10**, 1903338 (2020).

21. Liu, S. et al. Achieving high energy storage density and efficiency simultaneously in $Sr(Nb_{0.5}Al_{0.5})O_3$ modified $BiFeO_3$ based lead-free ceramics. *Chem. Eng. J.* **451**, 138916 (2023).

22. Yan, F. et al. Composition and structure optimized $BiFeO_3$-$SrTiO_3$ lead-free ceramics with ultrahigh energy storage performance. *Small* **18**, 2106515 (2022).

23. Guo, H. et al. Realizing high energy density in $BiFeO_3$-based ceramics capacitors via bandgap engineering and polarization optimization. *Chem. Eng. J.* **461**, 142071 (2023).

24. Chen, L. et al. Excellent energy storage and mechanical performance in hetero-structure $BaTiO_3$-based relaxors. *Chem. Eng. J.* **452**, 139222 (2023).

25. Wang, W. et al. Enhancement of energy storage performance in lead-free barium titanate-based relaxor ferroelectrics through a synergistic two-step strategy design. *Chem. Eng. J.* **434**, 134678 (2022).

26. Fu, J. et al. A highly polarizable concentrated dipole glass for ultrahigh energy storage. *Nat. Commun.* **15**, 7338, (2024).

27. Chen, X. et al. Ultrahigh energy density and efficiency of $BaTiO_3$-based ceramics via multiple design strategies. *Chem. Eng. J.* **467**, 143395 (2023).

28. Chen, L. et al. Local diverse polarization optimized comprehensive energy-storage performance in lead-free superparaelectrics. *Adv. Mater.* **34**, 2205787 (2022).

29. Hu, Q. et al. Achieve ultrahigh energy storage performance in $BaTiO_3$-$Bi(Mg_{1/2}Ti_{1/2})O_3$ relaxor ferroelectric ceramics via nano-scale polarization mismatch and reconstruction. *Nano Energy* **67**, 104264 (2020).

30. Wang, W. et al. Effective strategy to improve energy storage properties in lead-free $(Ba_{0.8}Sr_{0.2})TiO_3$-$Bi(Mg_{0.5}Zr_{0.5})O_3$ relaxor ferroelectric ceramics. *Chem. Eng. J.* **446**, 137389 (2022).

31. Sun, Z. et al. Superior capacitive energy-storage performance in Pb-free relaxors with a simple chemical composition. *J. Am. Chem. Soc.* **145**, 6194-6202 (2023).

32. Chen, L. et al. Near-zero energy consumption capacitors by controlling inhomogeneous polarization configuration. *Adv. Mater.* **36**, 2313285 (2024).

33. Li, D. et al. Improved energy storage properties achieved in (K, Na)NbO_3-based relaxor ferroelectric ceramics via a combinatorial optimization strategy. *Adv. Funct. Mater.* **32**, 2111776 (2022).

34. Zhang, M. et al. Significant increase in comprehensive energy storage performance of potassium sodium niobate-based ceramics via synergistic optimization strategy. *Energy Storage Mater.* **45**, 861-868 (2022).

35. Zhang, M. et al. Energy storage performance of $K_{0.5}Na_{0.5}NbO_3$-based ceramics modified by $Bi(Zn_{2/3}(Nb_{0.85}Ta_{0.15})_{1/3})O_3$. *Chem. Eng. J.* **425**, 131465 (2021).

36. Chen, L. et al. Giant energy-storage density with ultrahigh efficiency in lead-free relaxors via high-entropy design. *Nat. Commun.* **13**, 3089 (2022).

37. Xie, A. et al. Supercritical relaxor nanograined ferroelectrics for ultrahigh-energy-storage capacitors. *Adv. Mater.* **34**, 2204356 (2022).

38. Dai, Z. et al. A strategy for high performance of energy storage and transparency in KNN-based ferroelectric ceramics. *Chem. Eng. J.* **427**, 131959 (2022).

39. Chai, Q. et al. Superior energy storage properties and optical transparency in $K_{0.5}Na_{0.5}NbO_3$-based dielectric ceramics via multiple synergistic strategies. *Small* **19**, 2207464 (2023).

40. Lin, J. et al. Simultaneously achieving high performance of energy storage and transparency via A-site non-stoichiometric defect engineering in KNN-based ceramics. *Chem. Eng. J.* **444**, 136538 (2022).

41. Ren, X. et al. Regulation of energy density and efficiency in transparent ceramics by grain refinement. *Chem. Eng. J.* **390**, 124566 (2020).

42. Zhao, P. et al. $BaTiO_3$-$NaNbO_3$ energy storage ceramics with an ultrafast charge-discharge rate and temperature stable power density. *Microstructures* **3**, 2023002 (2023).

43. Zeng X. et al. Giant capacitive energy storage in high-entropy lead-free ceramics with temperature self-check. *Adv. Mater.* **36**, 2409059 (2024).

44. Peng, H. et al. High-entropy relaxor ferroelectric ceramics for ultrahigh energy storage. *Nat. Commun.* **15**, 5232 (2024).

45. Semenovskaya, S. et al. Development of ferroelectric mixed states in a random field of static defects. *J. Appl. Phys.* **83**, 5125-5136 (1998).

46. Zhou, M. et al. Strain, temperature, and electric-field effects on the phase transition and piezoelectric responses of $K_{0.5}Na_{0.5}NbO_3$ thin films. *J. Appl. Phys.* **123**, 154106 (2018).

47. Zhang, L. et al. Glass-glass transitions by means of an acceptor-donor percolating electric-dipole network. *Phys. Rev. Appl.* **8**, 054018 (2017).

48. Wang, J et al. Temperature-pressure phase diagram and ferroelectric properties of $BaTiO_3$ single crystal based on a modified Landau potential. *J. Appl. Phys.* **108**, 114105 (2010).
